# The Impact of Drug Trials With Financial Conflict of Interests on the Meta-analyses: A Meta-epidemiological Study

**DOI:** 10.34172/ijhpm.2021.162

**Published:** 2021-11-20

**Authors:** Na Zhang, Peijing Yan, Haitong Zhao, Lufang Feng, Xiajing Chu, Jingwen Li, Nan Chen, Kehu Yang, Xingrong Liu

**Affiliations:** ^1^Evidence Based Social Science Research Center, School of Public Health, Lanzhou University, Lanzhou, China.; ^2^Health Technology Assessment Center of Lanzhou University, School of Public Health, Lanzhou University, Lanzhou, China.; ^3^Evidence-Based Medicine Center, School of Basic Medical Sciences, Lanzhou University, Lanzhou, China.; ^4^Key Laboratory of Evidence Based Medicine and Knowledge Translation of Gansu Province, Lanzhou, China.; ^5^Department of Epidemiology and Health Statistics, West China School of Public Health and West China Fourth Hospital, Sichuan University, Chengdu, China.

**Keywords:** Cochrane, Meta-analyses, Drug Trials, Conflict of Interests, Meta-epidemiological

## Abstract

**Background:** To assess the impact of trials with potential financial conflict of interests (FCOIs) on evidence synthesis in meta-analyses (MAs).

**Methods:** A total of 96 MAs from the Cochrane Library about drug trials were investigated. The primary outcomes examined the proportion of conclusions that would change with the exclusion of trials with potential FCOIs. If the proportion of changed conclusions was below the non-inferiority margin of 10%, we considered that it was not inferior to include the trials with potential FCOIs in the MAs.

**Results:** Only 54.17% of MAs reported the funding sources of each included trial, and in 21.88% of MAs, the author-industry-related financial ties of each included trial were reported. When trials with FCOIs were excluded, the changed conclusions of effectiveness and major adverse events were 13.16% and 11.11%, respectively, and the I^2^ decreased by 13.56% and 10.09%, respectively. For serious adverse events, the exclusion of FCOIs trials did not lead to any change in conclusions; however, the I^2^ decreased by 24.24%. The impact of trials without reported FCOIs was also examined on evidence synthesis, and the results showed that the changed conclusions of effectiveness and major adverse events were 5.26% and 6.25%, respectively, indicating non-inferiority. However, the I^2^ increased by 13.60% and 12.37%, respectively.

**Conclusion:** In this meta-epidemiological study, we demonstrated that trials with FCOIs may not only influence the final outcome of MAs but may also increase the heterogeneity of results. It is suggested that all MAs fully report the FCOIs involved in evidence-based research and explore the impact of its FCOIs to better provide a more valuable reference for patients, clinicians, and policy-makers.

## Introduction

 Financial conflict of interests (FCOIs) were defined by the Institute of Medicine as “a set of conditions in which professional judgment concerning a primary interest (eg, patient’s welfare) tends to be unduly influenced by a secondary interest (eg, economic returns).”^1^ More than half of all the funding used for medical research is provided by the drug and device industry,^2^ and about two-thirds of drug trials are industry-funded (eg, providing drugs, placebo, researchers’ financial compensation).^3-5^ It has been reported that in the United States, industry invests more in medical research compared to the National Institutes of Health.^2,6^ It cannot be denied that the relationship between industry and research institutions has boomed, thereby promoting the advancement of medicine. However, a varied degree of entanglement of relationships among industry, academic institutions, and researchers has also emerged. Especially, drug trials with FCOIs may influence trial design, drug dosage, comparators, and promising results are more likely to be reported.^7^ In a previous survey, it was found that none of the 56 trials of non-steroidal anti-inflammatory drugs supported by the pharmaceutical factory presented results that were unfavorable to the company.^8^ In another survey, it was found that the conclusions recommended the experimental drug as the drug of choice as five times as often if the trial was funded by profit organizations, even after adjustment for the effect size.^9^ Nejstgaard et al found that FCOIs were associated with favorable recommendations of drugs and devices in clinical guidelines, advisory committee reports, opinion pieces, and narrative reviews.^10^

 High-quality systematic reviews (SRs) and meta-analyses (MAs) have been increasingly regarded as one of the key tools to ensure that the decision-making process is based on the best empirical evidence available.^11-13^ Inclusion of trials with FCOIs may result in distorted facts in SRs and Mas,^14^ which can lead to patients, clinicians, and policy-makers making suboptimal decisions.^12,15-17^ Thus, clinical research reporting standards and guidelines suggest and require active disclosure of the funding source between researchers and industries, both in drug trials and SRs and MAs.^18-20^ SRs and MAs published by Cochrane had a stricter FCOIs policy than most other journals,^21^ and the production process was rigorous. Therefore, they were widely regarded as the criteria for evaluating healthcare interventions.^22,23^ In 2012, the Cochrane Collaboration required Cochrane SRs and MAs to be published, to report funding sources and author-industry-related financial ties.^24,25^ In addition, in April 2018, the Governing Board of Cochrane updated the proposal to undertake a broader and stricter review of the Cochrane’s commercial sponsorship policy to ensure that the existing policy will lead to greater clarity, transparency, and a stricter approach to FCOIs.

 However, the Preferred Reporting Items for Systematic Reviews and Meta-analyses (PRISMA) statement, published in 2009 and 2020,^26,27^ did not address reporting of trial funding and author-industry-related financial ties from included trials. Furthermore, the International Committee of Medical Journal Editors (ICMJE) guidelines require authors of MAs to declare their own FCOIs but do not involve the reporting of study funding or author-related FCOIs of included trials.^28^ Roseman et al demonstrated that even MAs that were published in high-impact biomedical journals rarely reported the FCOIs of included trials.^17^ In this study, we investigated the extent to which Cochrane MAs of drug trials that were published between 2010 and 2019 reported funding sources and author-industry-related financial ties of included trials. Most importantly, we used the method of meta-epidemiological to assess the potential impact of FCOIs on the effect size of MAs and its conclusions.

## Methods

###  Data Sources and Search Strategy

 In this study, the Cochrane MAs of drug trials were searched between 2010 and 2019 using the Cochrane Library on June 12, 2020. The search terms were as follows: (meta analyses OR meta-analyses OR meta analysis OR meta-analysis OR metanalysis OR met-analysis OR met-analyses OR metanalyses OR data pooling) AND MeSH term “drug therapy.”

###  Eligibility Criteria

 Drugs were broadly defined as chemical substances and biologicals (including vaccines), but not nutritional supplements (eg, vitamins, probiotics), fluids, antiseptics, or medical devices without a drug component.^29^ Studies were considered to be eligible if they were MAs in which drug trials were evaluated that had: (*a*) at least one analyzed drug that was authorized by the US Food and Drug Administration at the time of publication; (*b*) a combination of drug and non-drug interventions (eg, cognitive therapy, psychotherapy); (*c*) synthesized results from ≥2 trials; (*d*) evaluated the effectiveness or adverse events of a drug or class of drugs. MAs in which only different dosages, the dosing interval, or method of administration were assessed, were excluded from the study.

###  Selection Process

 Two researchers (NZ and LFF) independently screened the title and abstract of MAs according to the inclusion and exclusion criteria. Disagreements were resolved through discussion or by consulting a third investigator (PJY). Subsequently, among the MAs that met the inclusion criteria, 100 MAs were selected by two-stage stratified random sampling.

 Two-stage stratified random sampling was performed as follows: (*a*) the selected studies were stratified into ten levels based on the year of publication; (*b*) studies were selected at each level in equal proportion by computer randomization. Finally, studies included to be analyzed were determined by reading the full text. Figure S1 shows the flow of ascertainment and sampling (See Supplementary file 1).

###  Data Extraction

 Two researchers (NZ and XJC) independently extracted the following items: (*a*) MAs: first author, year, country/region (belonging to the first affiliation), name of disease, population, intervention and comparator, study design, outcomes and adverse events, funding sources, disclosure statements, author-industry-related financial ties and whether the risk of bias evaluation related to FCOIs was conducted as well as its reasons why; (*b*) trials included in MAs: funding sources, disclosure statements, and author-industry-related financial ties.

###  Data Management

 MAs or trials with potential FCOIs were considered if the funding sources were derived from the pharmaceutical industry, pharmaceutical industry and non-industry, or when participating authors had financial ties with the pharmaceutical industry.

 The funding sources were classified as follows: (*a*) funded by the pharmaceutical industry (eg, provision drugs and placebos directly); (*b*) funded by non-profit organization (eg, national funding supporting institutions); (*c*) funded by pharmaceutical industry and non-industry; (*d*) funding sources not known (not funded by any organization or institution); (*e*) funding sources not reported (funding information not disclosed).^30^ The author-industry-related financial ties of MAs were defined according to the ICMJE uniform disclosure form, including current or former board membership, current or former consultancy work, current or former industry employment, expert testimony, industry grants (issued or pending), payment for lectures including service related to speakers bureaus, payment for manuscript preparation, patents (planned, pending, or issued), royalties, payment for the development of educational presentations, stock or stock options, travel reimbursement, or other items associated with industry-related relations, as disclosed in the MAs.^28^ If MAs or trials did not contain disclosure statements, the author-industry-related financial ties were coded as not reported, and if the author reported that they did not have any relationship with industry, the author-industry-related financial ties were considered as none known.

 The comparison arms were divided into the following: (*a*) active drug only; (*b*) placebo/ no intervention; (*c*) active drug combined with placebo/no intervention according to the intervention category of the control group. An “active drug” was defined as an alternative drug that had the same efficacy as the drug to be evaluated; “placebo” referred to physical properties (eg, appearance, size, color, dosage form, weight, taste and smell) that were similar to those of the tested drug but did not contain the effective ingredients of the tested drug; “no intervention” referred to a control group that did not receive any treatment.

###  Data Analysis

 After excluding the trials with FCOIs, we reperformed MAs employed the same statistical tool (eg, Stata 15.0, Stata Corporation, 2017; RevMan 5.4, The Cochrane Collaboration, 2020) and model as the original MAs. We next evaluated whether excluding trials with FCOIs would result in no trials available for pooled analysis in the MAs. We mainly analyzed the effectiveness, major adverse events, and severe adverse events contained in MAs. In the event that multiple primary outcomes could all be recalculated after excluding the trials with FCOIs, the primary outcome with the most included trials was selected for analysis. If primary outcomes could not be recalculated and analyzed after removing trials with FCOIs, a secondary outcome with the most included trials was selected for analysis. The same criteria were used for major adverse events and severe adverse events. A two-tailed *P* < .05 was considered statistically significant.

 The primary outcome of this study was the proportion of changed conclusions based on excluding the drug trials with FCOIs compared to the original conclusions based on the evidence of comprehensive funding sources in MAs. We defined “changed conclusion” as a conclusion that reverted its direction (effective became invalid, or vice versa). If the conclusions were identical to those in the MAs, we deemed that the conclusions had not changed, even if the certainty was reduced. Secondary outcomes included a change in heterogeneity (statistical heterogeneity was quantified and represented by the I^2^).^31^ In addition, we explored the impact of trials without reported FCOIs on the synthesis of MAs. Excel 2019 was employed to compare the percentages of changed conclusions and the percentages of heterogeneity (I^2^) between recalculated MAs and original MAs.

 The non-inferiority design is based on the prior effect and safety information to test the effect of two methods, and its results and conclusions are more reliable.^32^ The threshold for the non-inferiority margin was set to 10%, and was based on the finding of an international survey which showed that decision-makers in healthcare are willing to accept a 10% incremental risk of getting an incorrect result.^33^ In this study, if the proportion of changed conclusions was below the non-inferiority margin of 10%, we considered including trials with FCOIs in MAs as a non-inferiority.

###  Subgroup Analysis and Sensitivity Analysis

 Subgroup analysis was conducted to explore the impact of funding sources and author-industry-related financial ties on the synthesis of MAs.

 Any recalculated MA that involved only one trial to test the robustness of our findings was excluded from the study.

## Results

###  Identified Studies

 Our initial search yielded 1227 studies, 169 of which were trials, and the remaining 1058 SRs and MAs were selected for further evaluation through reading the title and abstract. A total of 613 MAs met the inclusion criteria (Figure S1). Of these eligible MAs, 19 were published in 2011, which was the year with the least included MAs, while 2015 with 81 MAs was the year with the most. Next, 100 MAs were selected by two-stage stratified random sampling based on the year of publication, while four MAs^34-37^ were excluded due to the study design, which appeared to be SRs after reading the full text. Finally, 96 MAs were included in this study. The smallest number of MAs included in this study were published in 2011 (n = 3), while the most were published in 2014, 2015, 2016 and 2017, including 13 MAs per year published.

###  Study Characteristics

 The number of included trials of 96 Cochrane MAs ranged from 2 to 196 (Table 1). In addition, 30.21% (29/96) of MAs were produced in the Britain; 14.58% (14/96) focused on diseases of the digestive system; 68.75% (66/96) of the participants’ age in MAs were at any stage; 44.79% (43/96) of comparison arms were active drugs combined with placebo/no intervention; 87.50% (84/96) of the MAs included randomized controlled trials (RCTs) only.

**Table 1:** Summary Characteristics of Included 96 Meta-analyses

**Characteristics**	**No. (%) or Mean (Minimum-Maximum)**
	**N = 96 (All MAs)**
Population	
Adults	24 (25.00)
Children	3 (3.13)
Neonates	3 (3.13)
Any age	66 (68.75)
Type of comparison arm(^s^)	
Active drug	25 (26.04)
Placebo/no intervention	28 (29.27)
Active drug combined with placebo/ no intervention	43 (44.79)
Classification of diseases^a^	
Diseases of the digestive system	14 (14.58)
Diseases of the nervous system	12 (12.50)
Diseases of the respiratory system	12 (12.50)
Mental, behavioral or neurodevelopmental disorders	9 (9.38)
Diseases of the genitourinary system	8 (8.33)
Neoplasms	7 (7.29)
Pregnancy, childbirth or the puerperium	7 (7.29)
Certain infectious or parasitic diseases	6 (6.25)
Endocrine, nutritional or metabolic diseases	6 (6.25)
Others	15 (15.63)
Number of included trials	19 (2-196)
Study design of included trials	
RCTs only	84 (87.50)
RCTs + at least one more other study designs	12 (12.50)
Reporting on funding sources of trials included in MAs	52 (54.17)
Reporting on author-industry-related financial ties of trials included in MAs	21 (21.88)
Funding sources of MAs	
Pharmaceutical industry	0 (0.00)
Pharmaceutical industry and non-profit organization	16 (16.67)
Non-profit organization	66 (68.75)
None known	14 (14.58)
NR	0 (0.00)
Author-industry-related financial ties of MAs	
Yes	15 (15.63)
None known	81 (84.37)
NR	0 (0.00)
MAs authors’ working countries	
Britain	29 (30.21)
Canada	14 (14.58)
Australia	9 (9.38)
China	6 (6.25)
America	5 (5.21)
Others	33 (34.30)

Abbreviations: Mas, meta-analyses; RCTs, randomized controlled trials; NR, not reported. ^a^ Disease classification was based on International Classification of Diseases, 11th edition.

###  Reporting of Financial Conflict of Interests 

 All 96 included MAs reported FCOIs (Table 1). Of these, 68.75% (66/96) were funded by non-profit organizations, 16.67% (16/96) were funded by the pharmaceutical industry and non-profit organizations, and 14.58% (14/96) had no reportable FCOIs. Furthermore, 15.63% (15/96) of MAs had author-industry-related financial ties, and 84.37% were described as “none known.” A total of 54.17% (52/96) of MAs reported the funding sources of each included trials, and 21.88% (21/96) reported author-industry-related financial ties of the included trials. However, only 1.04% (1/96) of MAs^38^ performed subgroup analysis based on the FCOIs, and 6.25% (6/96) planned to do further analysis, which could not be carried out due to an insufficient dataset.

 As shown in Table 2, 23.44% (226/964) of the trials in recalculated MAs were funded by the pharmaceutical industry, 3.73% (36/964) were funded by industry and non-profit organizations, 17.53% (169/964) were funded by non-profit organizations, 40.35% (389/964) were identified as “none known,” and in 14.94% (144/964), funding sources were not reported. As for author-industry-related financial ties, 25.31% (244/964) demonstrated author-industry-related financial ties, 55.19% (532/964) were identified as “none known,” and in 19.50% (188/964), author-industry-related financial ties were not reported. Details were listed in Table S1.

**Table 2:** The Financial Conflict of Interests of Included 964 Trials in Recalculated 48 Meta-analyses

**Type of FCOIs**	**No. (%) **
Funding sources	
Pharmaceutical industry	226 (23.44)
Pharmaceutical industry and non-profit organization	36 (3.73)
Non-profit organization	169 (17.53)
None known	389 (40.35)
NR	144 (14.94)
Author-industry financial ties	
Existing author-industry financial ties	244 (25.31)
None known	532 (55.19)
NR	188 (19.50)

Abbreviations: FCOIs, financial conflict of interests; NR, not reported.

###  Impact on Conclusions and Heterogeneity

####  Excluding Trials With Financial Conflict of Interests

**Figure 1:**
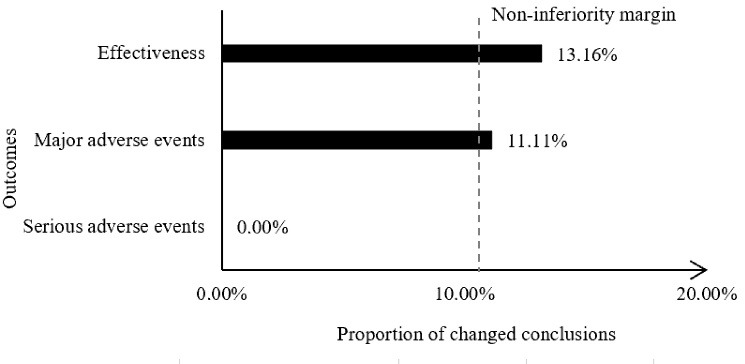
Proportion of Changed Conclusions After Excluding the Trials With Financial Conflict of Interests.

 For effectiveness, five out of 48 MAs in which trials with FCOIs were excluded, showed opposite conclusions (13.16%) (Figure 1). In addition, most of them (4/5) changed from statistical significance to no statistical significance (Table S2). When one recalculated MA that only included one trails was excluded, non-inferiority (10.53%) was still not achieved. In terms of heterogeneity in effectiveness, the I^2^ decreased by 13.56% (Table 3).

**Table 3:** Changes in Heterogeneity

**Items**	**I** ^2^ ** (%)**
Overall analysis	
Excluding the trials with FCOIs	
Effectiveness	-13.56
Major adverse events	-10.19
Serious adverse events	-24.24
Trials without reported FCOIs	
Effectiveness	+13.60
Major adverse events	+12.37
Serious adverse events	- 4.35
Subgroup analysis	
FCOIs from funding sources	
Effectiveness	-21.23
Major adverse events	+9.37
Serious adverse events	-17.14
FCOIs from author-industry-related financial ties	
Effectiveness	+8.60
Major adverse events	-12.85
FCOIs from funding sources and author-industry-related financial ties	
Effectiveness	-15.32
Major adverse events	-22.69
Serious adverse events	-

Abbreviation: FCOIs, financial conflict of interests.

 For major adverse events, three out of 27 MAs in which trials with FCOIs were excluded, showed opposite conclusions (11.11%) that all changed from statistical significance to no statistical significance (Table S3). The I^2^ decreased by 10.09%, which was upper the non-inferiority margin of 10%. For serious adverse events, the exclusion of trials that reported FCOIs did not lead to any change in conclusions (Table S4), however, the I^2^ decreased by 24.24%. The results of sensitivity analysis showed that, excluding recalculated MAs that included only one trial did not lead to any change.

####  Trials Without Reported Financial Conflict of Interests

**Figure 2:**
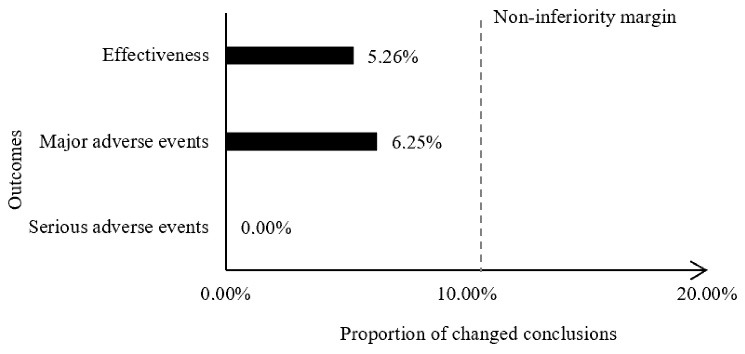
Proportion of Changed Conclusions When the Trials Without Reported Financial Conflict of Interests.

 One out of 19 reperformed MAs showed opposite conclusions (5.26%) (Figure 2), which changed from not statistically significant to statistically significant (Table S5). For heterogeneity, the I^2^ increased by 13.60% (Table 3).

 For major adverse events, one out of 16 MAs showed opposite conclusions (6.25%), indicating non-inferiority, and all changed from statistical significance to not statistically significant (Table S6), however, the I^2^ increased by 12.37%. For serious adverse events, the exclusion of trials with FCOIs did not lead to any change in conclusions (Table S7). The I^2^ decreased by 4.35%, indicating non-inferiority. The results of sensitivity analysis showed that, excluding the recalculated MAs that included only one trial did not lead to any change.

###  Subgroup Analysis

####  Type of Financial Conflict of Interests Was Funding Sources

**Figure 3:**
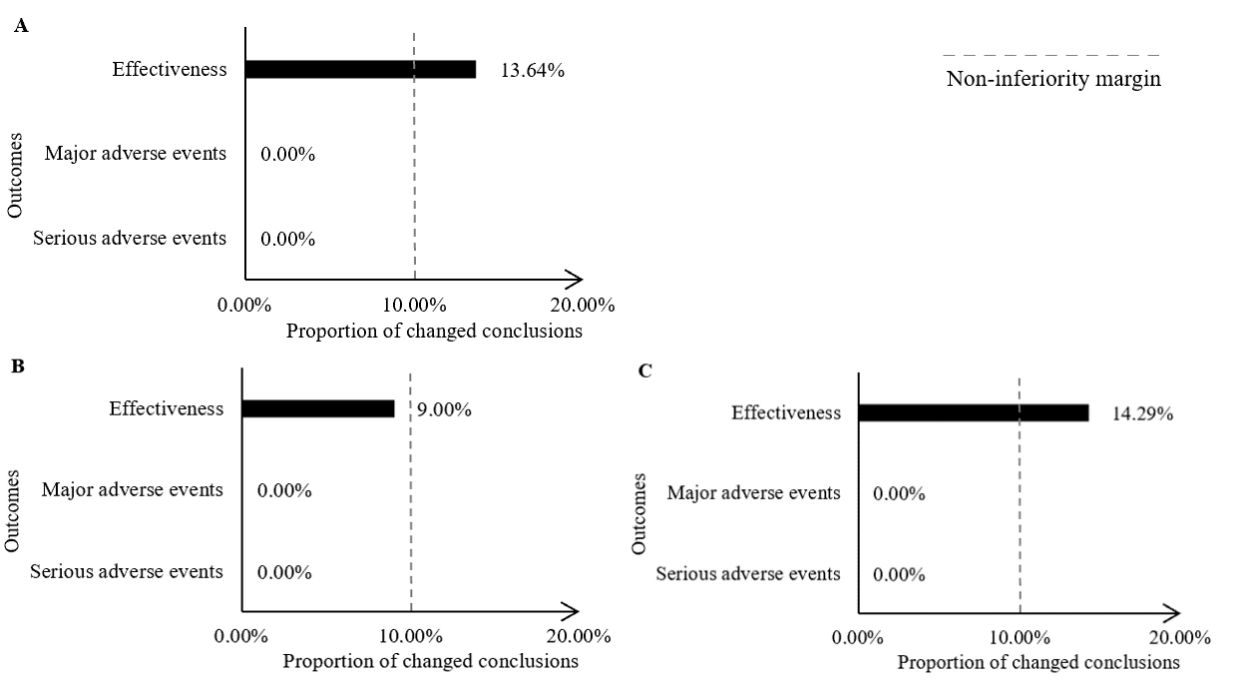
Subgroup Analysis: Proportion of Changed Conclusions.

 For effectiveness, when excluding trials that received funding from the pharmaceutical industry, three out of 22 MAs showed opposite conclusions (13.64%) (Figure 3). For heterogeneity, the I^2^ decreased by 21.23% (Table 3).

 For major adverse events, none of the conclusions of the recalculated MAs showed opposite conclusions, and the I^2^ increased by 9.37%. For serious adverse events, none of the conclusions of the recalculated MA changed, however, the I^2^ decreased by 17.14%.

####  Type of Financial Conflict of Interests Was Author-Industry-Related Financial Ties

 For effectiveness, for excluding trials with author-industry-related financial ties, one out of 9 MAs showed opposite conclusions (9.00%) (Figure 3), and the I^2^ increased by 8.60% (Table 3).

 For major adverse events and serious adverse events, none of the conclusions of recalculated MAs changed, and the I^2^ increased by 9.37%.

####  Type of Financial Conflict of Interests Were Funding Sources and Author-Industry-Related Financial Ties

 In terms of effectiveness, when trials with both funding sources and author-industry-related financial ties were excluded, one out of seven MAs showed opposite conclusions (14.29%) (Figure 3), and the I^2^ decreased by 15.32% (Table 3).

 For major adverse events, none of the conclusions of the recalculated MAs showed opposite conclusions, and the I^2^ decreased by 22.69%. For serious adverse events, none of the conclusions of the recalculated MAs changed.

 The results of sensitivity analysis showed that, excluding recalculated MAs that included only one trial did not lead to any change.

## Discussion

 All 96 MAs reported FCOIs. However, only 54.17% of MAs reported the funding sources of each included trial, and in 21.88% of MAs, the author-industry-related financial ties of included trials were reported. Moreover, in only 1.04% of MAs subgroup analysis was performed based on the FCOIs, and in 67.37% of MAs it was not clear from the text why subgroup analysis or sensitivity on FCOIs was not performed. When trials with FCOIs were excluded, the changed conclusions of effectiveness and major adverse events did not achieve non-inferiority, and the heterogeneity increased by more than 10%. For serious adverse events, the exclusion of FCOIs trials did not lead to any change in conclusions; however, the heterogeneity decreased by more than 10%. The impact of trials without reported FCOIs on evidence synthesis was also examined, and the results showed that the changed conclusions of effectiveness and major adverse events achieved non-inferiority. However, the heterogeneity increased by more than 10%. Subgroup analysis showed that, when the type of FCOIs included funding sources, the proportion of changed conclusions of effectiveness did not achieve non-inferiority. When the type of FCOIs was author-industry-related financial ties, the changed conclusions and heterogeneity achieved non-inferiority.

 The reporting of opaque FCOIs may affect scientific judgment, lose objectivity and publicity, and may greatly impact decision-making. Therefore, in 2009, the ICMJE formulated the Uniform Requirements for Manuscripts Submitted to the Biomedical Journal, which mainly included ethical issues related to research implementation, reporting etc.^28^ In 2010, the ICMJE renewed this requirement.^30^ In this renewal, the most important issue was the reporting of FCOIs in journal publication. A unified tabulation of FCOIs disclosure was formulated to settle this problem. In 2012, the Cochrane Collaboration began to require that funding sources of trials and FCOIs of authors of included trials be reported in a table named “characteristics of included studies” of all Cochrane SRs and MAs^25^; this is a mandatory requirement. However, the results of this study showed that in only 54.17% of MAs the FCOIs of the included primary studies were reported and listed in detail, whereas in only 21.88% of MAs, author-industry-related financial ties of included trials were reported. In a recent study, it was demonstrated that the reporting of non-Cochrane SRs and MAs on the financial relationship of the author-industry was only 1%,^39^ which is far from acceptable.

 The results of this study showed that excluding studies with FCOIs resulted in a 13.16% change in conclusions on the effectiveness, and most changed from statistically significant to not statistically significant (80.00%). In addition, the impact of trials without reported FCOIs on evidence synthesis was examined. The results showed that the changed conclusions of effectiveness and major adverse events were 5.26% and 6.25%, respectively, thereby indicating non-inferiority. However, the I^2^ increased by 13.60% and 12.37%, respectively. Taken together, the data showed that trials with FCOIs may not only influence the final outcome of MAs but may also increase the heterogeneity of the research results.

 To reduce the impact of factory-funded trials on heterogeneity, it is important to conduct subgroup or sensitivity analysis of FCOIs for included trials. However, our results showed that this was only achieved for 1.04% of the studies, and in 67.37% of the Cochrane MAs, subgroup analysis or sensitivity analysis was not conducted, and no explanation was given. Therefore, we suggest that: (*a*) drug trials should undertake clinical trial registries to reduce bias; (*b*) the pharmaceutical industry should support investigator-initiated research based on scientific interest; (*c*) we should adequately report the FCOIs in included studies and discuss funding sources by subgroup analysis, sensitivity analysis or some other way in meta-analysis to provide more valuable advice to decision makers and physicians.

###  Limitations

 Our study has several limitations. Firstly, for each MA, we only selected one primary effect and adverse events that could be used for analysis but we did not analyze the change of all conclusions. However, the change of other conclusions may also affect the overall effect evaluation of drugs. Secondly, in this study, only statistical heterogeneity was explored, and other sources of heterogeneity (eg, clinical heterogeneity) were not investigated. Thirdly, this study was only based on drug trials published by Cochrane MAs which methodological quality and reporting quality are relatively high.^25^ Therefore, interpretation of the results should be cautious. Fourthly, in this study, we only analyzed the revealed FCOIs, but did not analyze the undisclosed conflicts of interest and non-FCOIs for the lack of data. However, these two types of conflicts of interest may also have a certain impact on judgment.^13,40,41^ Fifthly, we did not have a published protocol to document the decisions that were made throughout the study. Finally, studies included in the analysis were selected by random sampling, which may result in this study not being repeatable.

## Conclusion

 In summary, this meta-epidemiological study demonstrated that trials with FCOIs may not only influence the final outcome of MAs but may also increase the heterogeneity of the research results. The Cochrane Collaboration is a recognized leader in the establishment of methodology for the conduct and reporting of evidence-based reviews. In this study, however, we found that in 45.83% of Cochrane MAs of drug trials, the FCOIs of included trials were not reported, and in 78.12% the author-industry-related financial ties of included trials were not reported. Therefore, to better provide valuable references for doctors, patients, and policy makers, we suggest that all MAs should enclose all FCOIs involved in evidence-based research and explore the impact of its FCOIs through subgroup analysis or sensitivity analysis.

## Ethical issues

 Not applicable.

## Competing interests

 Authors declare that they have no competing interests.

## Authors’ contributions

 NZ: Conceptualization, methodology, formal analysis, resources, writing-original draft, writing - review & editing. PY: Software, validation, formal analysis, investigation, resources, writing - original draft. HZ: Investigation, resources, writing-original draft, draft repairing. LF: Formal analysis, data curation, investigation, resources, writing-original draft. XC: Formal analysis, investigation, resources, writing-original draft. JL: Data curation, resources, writing-original draft. NC: Data curation, writing-original draft. XL: Conceptualization, data curation, writing - review & editing, supervision, funding acquisition, draft repairing. KY: Conceptualization, data curation, writing - review & editing, supervision, funding acquisition, draft repairing.

## Funding

 This study was supported by the Key Research and Development Program of “Belt and Road Initiative,” Lanzhou University.

## Supplementary files

Supplementary file 1 contains Figure S1 and Tables S1-S7.
