# Supplementary material for: The Impact of Drug Trials With Financial Conflict of Interests on the Meta-analyses: A Meta-epidemiological Study
Source: Int J Health Policy Manag. 2021 Nov 20;11(10):2038–45. doi: 10.34172/ijhpm.2021.162 (PMC9808270; doi:10.34172/ijhpm.2021.162)
Supplement: Supplementary file 1 — contains Figure S1 and Tables S1-S7. [file ijhpm-11-2038-s001.pdf]

Article title: The Impact of Drug Trials With Financial Conflict of Interests on the Meta-analyses: A Meta-epidemiological Study

Journal name: International Journal of Health Policy and Management (IJHPM)

Authors' information: Na Zhang[1,2,3,4] ¶, Peijing Yan[5] ¶, Haitong Zhao[1,2,3,4], Lufang Feng[1,2,3,4], Xiajing Chu[1,2,3,4],Jingwen Li[1,2,3,4], Nan Chen[1,2,3,4], Kehu Yang[1,2,3,4]*, Xingrong Liu[1]*

[1]Evidence Based Social Science Research Center, School of Public Health, Lanzhou University, Lanzhou, China.

[2]Health Technology Assessment Center of Lanzhou University, School of Public Health, Lanzhou University, Lanzhou, China.

[3]Evidence-Based Medicine Center, School of Basic Medical Sciences, Lanzhou University, Lanzhou, China.

[4]Key Laboratory of Evidence Based Medicine and Knowledge Translation of Gansu Province, Lanzhou, China.

[5]Department of Epidemiology and Health Statistics, West China School of Public Health and West China Fourth Hospital, Sichuan University, Chengdu, China

¶Both authors contributed equally to this paper.

(*Corresponding author: kehuyangebm2006@126.com & liuxr@lzu.edu.cn)

Supplementary file 1

Table S1. FCOIs of 48 recalculated MAs.

Study	Trials included MA	Funding source of MA	Author-industry financial ties of MA	Funding source of included trials (n = 964)					Author-industry financial ties of included trials (n = 964)		
				No. pharmaceutical industry	No. pharmaceutical industry and non-profit organization	No. non-profit organization	No. NR	No. none known	No. existing author-industry financial ties	No. NR	No. none known
Brand, 2010	9	None known	None known	1	0	3	2	3	1	1	7
Marshall, 2010	38	None known	None known	17	4	3	6	8	12	9	17
Stowe, 2010	44	None known	Yes	17	0	12	0	15	15	0	29
Martin-Hirsch, 2011	7	Non-profit organization	None known	2	0	2	0	3	1	0	6
Zani, 2011	19	None known	None known	6	0	3	0	10	7	0	12
Chauhan, 2012	65	Non-profit organization	Yes	0	0	13	25	27	0	29	36
Sinclair, 2012	8	Non-profit organization	None known	1	0	2	0	5	0	0	8
Nair, 2012	17	Non-profit organization	None known	3	1	3	4	6	2	6	9
Lodi, 2012	18	Non-profit organization	None known	7	0	1	3	7	9	6	3
Rudic, 2012	16	Non-profit organization	None known	4	1	3	2	6	4	6	6
Anderson-James, 2013	2	Pharmaceutical industry and non-profit organization	None known	1	0	1	0	0	0	0	2
Brown, 2013	10	Non-profit organization	Yes	0	0	2	4	4	0	3	7
Sharma, 2013	10	Non-profit organization	None known	4	0	2	0	4	4	0	6
Flenady, 2013	14	Non-profit organization	None known	3	1	1	2	7	3	2	9
Massel, 2013	13	Non-profit organization	None known	2	0	2	0	9	3	0	10
Sampson, 2013	17	Non-profit organization	None known	4	1	6	0	6	8	0	9
Prasad, 2014	18	None known	None known	1	0	0	0	1	0	1	1

Zani, 2014	27	Non-profit organization	None known	13	4	6	0	4	8	0	19
Clarke, 2014	6	Non-profit organization	None known	2	1	1	1	1	1	2	3
Cury Martins, 2015	20	Pharmaceutical industry and non-profit organization	None known	15	0	2	0	3	18	0	2
Dold, 2015	63	Pharmaceutical industry and non-profit organization	Yes	0	0	12	17	34	0	24	39
Sherlock, 2015	6	Non-profit organization	Yes	1	0	1	1	3	1	3	2
Tanner, 2015	15	Pharmaceutical industry and non-profit organization	None known	9	1	1	0	4	7	0	8
Chande, 2016	13	Non-profit organization	Yes	5	0	3	0	5	4	0	9
Conway, 2016	30	Non-profit organization	None known	16	2	2	3	7	14	5	11
Nielsen, 2016	6	Non-profit organization	None known	2	0	1	1	2	2	1	3
Rattehalli, 2016	15	Non-profit organization	None known	3	0	4	0	8	4	0	11
Mbuagbaw, 2016	12	Pharmaceutical industry and non-profit organization	None known	2	0	3	0	7	2	0	10
Wang, 2016	41	Non-profit organization	Yes	0	0	9	14	18	0	21	20
Zhu, 2016	6	Pharmaceutical industry and non-profit organization	None known	0	0	2	2	2	0	2	4
Derry, 2017	6	Non-profit organization	Yes	2	1	0	0	3	1	0	5
Knightly, 2017	6	Non-profit organization	None known	1	0	0	1	3	0	2	4
Onland, 2017	8	None known	None known	1	0	1	0	6	2	0	4
Robertson, 2017	6	Non-profit organization	None known	1	0	0	1	4	1	2	3
Shah, 2017	2	None known	None known	1	0	1	0	0	0	0	2
Tickell-Painter, 2017	6	Non-profit organization	None known	1	0	1	0	4	1	0	5
Verbeeck, 2017	20	Non-profit organization	None known	4	2	5	2	7	7	3	10
Zaman, 2017	196	Non-profit organization	None known	52	11	29	35	69	74	38	84

Gallos, 2018	8	Pharmaceutical industry and non-profit organization	Yes	0	0	2	2	4	0	2	6
Mehta, 2018	12	Non-profit organization	None known	0	0	1	3	8	0	4	8
Panebianco, 2018	10	Non-profit organization	None known	1	0	3	0	6	1	0	9
Romantsik, 2018	2	Non-profit organization	None known	1	0	1	0	0	0	0	2
Sliwka, 2018	13	Non-profit organization	None known	2	0	3	0	8	3	0	10
Tenforde, 2018	12	None known	None known	2	2	4	0	4	3	0	9
Altmann, 2019	14	Non-profit organization	None known	1	1	2	2	8	2	5	7
Gagyor, 2019	16	Pharmaceutical industry and non-profit organization	None known	3	1	3	2	7	3	5	8
Solomon, 2019	22	Pharmaceutical industry and non-profit organization	None known	4	1	3	3	11	4	1	17
Toupin April, 2019	9	None known	None known	1	0	3	2	3	1	1	7

FCOIs: financial conflict of interests; MAs: meta-analyses; NR: not reported.

Table S2. Impact of excluding the trials with FCOIs on the conclusion of effectiveness.

Study	Statistical Method	Effect Estimate		*P* value		I^2	
		Original	Recalculated	Original	Recalculated	Original	Recalculated
Brand, 2010	RR (fixed model)	0.57 (0.34, 0.94)	0.45 (0.22, 0.92)	.028	.030	0	10
Marshall, 2010	OR (random model)	1.65 (1.11, 2.45)	0.15 (0.08, 0.22)	.010	< .001	0	0
Stowe, 2010	MD (fixed model)	-1.54 (-1.83, -1.26)	-1.10 (-1.33, -0.86)	< .001	< .001	36	42
Martin-Hirsch, 2011	RR (random model)	0.86 (0.56, 1.32)	0.64 (0.27, 0.97)	.490	.300	79	90
Zani, 2011	RR (fixed model)	0.25 (0.15, 0.42)	0.22 (0.12, 0.41)	< .001	< .001	0	0
Sinclair, 2012	RR (fixed model)	0.71 (0.62, 0.80)	0.71 (0.11, 0.98)	< .001	< .001	0	15
Nair, 2012	MD (random model)	-0.36 (-4.09, 3.38)	0.92 (0.39, 3.32)	.850	.530	31	0
Lodi, 2012	RR (random model)	0.29 (0.16, 0.50)	0.20 (0.09, 0.74)	< .001	< .001	28	0

Rudic, 2012	RR (fixed model)	0.97 (0.67, 1.42)	1.14 (0.52, 2.67)	.890	.810	0	83
Anderson-James, 2013	OR (fixed model)	0.61 (0.24, 1.55)	0.84 (0.63, 0.90)	.300	.810	0	0
Sharma, 2013	RR (random model)	1.13 (1.01, 1.28)	1.13 (0.96, 1.32)	.040	.150*	82	87
Flenady, 2013	RR (fixed model)	1.22 (0.88, 1.69)	2.11 (0.78, 5.73)	.240	.140	0	0
Massel, 2013	OR (fixed model)	0.43 (0.32, 0.59)	0.45 (0.33, 0.62)	< .001	< .001	0	0
Sampson, 2013	RR (fixed model)	2.46 (1.70, 3.54)	2.09 (0.44, 3.01)	< .001	.007	0	49
Prasad, 2014	RR (fixed model)	0.43 (0.30, 0.62)	0.46 (0.29, 0.72)	< .001	< .001	0	-
Zani, 2014	RR (fixed model)	0.41 (0.21, 0.80)	0.38 (0.19, 0.76)	.010	.006	49	55
Clarke, 2014	RR (fixed model)	5.60 (4.02, 7.81)	5.46 (3.61, 8.24)	< .001	< .001	50	0
Cury Martins, 2015	RR (fixed model)	0.82 (0.72, 0.92)	0.80 (0.66, 0.98)	.001	.030	0	-
Tanner, 2015	OR (random model)	0.40 (0.07, 2.25)	0.67 (0.26, 3.09)	.300	.420	0	0
Chande, 2016	RR (random model)	1.53 (1.05, 2.22)	1.75 (0.58, 5.27)	.026	.320*	64	86
Nielsen, 2016	RR (random model)	0.69 (0.39, 1.22)	1.24 (0.83, 1.85)	.200	.300	68	23
Rattehalli, 2016	RR (random model)	0.69 (0.62, 0.78)	0.65 (0.55, 0.76)	< .001	< .001	25	0
Mbuagbaw, 2016	RR (random model)	1.04 (0.99, 1.09)	1.04 (0.51, 1.74)	.110	.230	0	0
Derry, 2017	RD (fixed model)	-0.04 (-0.16, 0.08)	0.00 (-0.22, 0.22)	.560	1.000	0	-
Knightly, 2017	MD (fixed model)	3.34 (-1.58, 8.26)	6.02 (0.48, 0.86)	.180	.040[#]	43	31
Onland, 2017	RR (fixed model)	0.60 (0.49, 0.98)	0.64 (0.35, 0.94)	< .001	.003	86	0
Robertson, 2017	Peto OR (fixed model)	0.69 (0.56, 0.93)	1.08 (0.07, 1.34)	.041	.070*	0	-
Shah, 2017	RR (random model)	0.83 (0.56, 1.23)	0.89 (0.32, 1.17)	.360	.400	0	62
Tickell-Painter, 2017	RR (random model)	0.09 (0.04, 0.19)	0.06 (0.07, 0.48)	< .001	< .001	53	0
Zaman, 2017	RR (fixed model)	1.13 (0.83, 1.54)	0.92 (0.04, 1.89)	.450	.740	18	0
Gallos, 2018	RR (random model)	1.26 (1.11, 1.43)	0.94 (0.60, 1.49)	< .001	.800*	0	0
Romantsik, 2018	RR (fixed model)	7.48 (4.91, 11.38)	7.35 (4.65, 11.64)	< .001	< .001	27	35
Sliwka, 2018	OR (random model)	6.12 (0.73, 51.24)	5.20 (0.25, 109.37)	.090	.290	0	-
Tenforde, 2018	RR (random model)	0.90 (0.45, 1.77)	1.13 (0.34, 0.94)	.750	.810	24	51

Altmann, 2019	OR (random model)	1.16 (0.71, 1.91)	0.40 (0.04, 3.94)	.550	.430	0	-
Gagyor, 2019	RR (random model)	0.54 (0.38, 0.77)	0.51 (0.34, 0.76)	< .001	< .001	40	28
Solomon, 2019	RR (fixed model)	0.57 (0.39, 0.84)	0.54 (0.36, 0.80)	.005	.003	23	24
Toupin April, 2019	SMD (fixed model)	-0.25 (-0.23, -0.19)	-0.24 (-0.26, -0.15)	< .001	< .001	0	0

FCOIs: financial conflict of interests; RR: risk ratio; OR: odds ratio; MD: mean difference; Peto OR: Peto odds ratio; SMD: standardized mean difference.

*: The conclusion changed from statistical significance to no statistical significance.

#: The conclusion changed from no statistical significance to statistical significance.

Table S3. Impact of excluding the trials with FCOIs on the conclusion of major adverse events.

Study	Statistical Method	Effect Estimate		*P* value		I^2	
		Original	Recalculated	Original	Recalculated	Original	Recalculated
Stowe, 2010	Peto OR (fixed model)	1.20 (1.00, 1.43)	1.13 (0.89, 1.43)	.046	.330*	37	43
Lodi, 2012	RR (random model)	1.98 (1.10, 3.59)	1.49 (1.16, 8.64)	.023	.031	11	1
Rudic, 2012	RR (fixed model)	0.87 (0.68, 1.12)	0.75 (0.46, 1.24)	.280	.270	23	0
Flenady, 2013	RR (fixed model)	1.32 (0.92, 1.89)	1.53 (0.91, 2.57)	.130	.110	0	0
Prasad, 2014	RR (fixed model)	0.43 (0.30, 0.62)	0.43 (0.30, 0.62)	< .001	< .001	48	-
Zani, 2014	RR (fixed model)	1.20 (0.59, 2.42)	1.29 (0.61, 2.68)	.620	.490	0	0
Clarke, 2014	RR (fixed model)	0.91 (0.74, 1.12)	0.81 (0.63, 1.05)	.370	.110	0	0
Dold, 2015	RR (random model)	1.10 (0.31, 3.91)	1.10 (0.31, 3.91)	.890	.890	0	30
Sherlock, 2015	RR (fixed model)	1.09 (0.95, 1.26)	1.03 (0.86, 1.22)	.220	.770	54	51
Nielsen, 2016	RR (random model)	1.10 (0.64, 1.91)	1.13 (0.64, 1.97)	.720	.680	52	-
Rattehalli, 2016	RR (random model)	1.56 (1.13, 2.15)	1.44 (0.97, 2.94)	.007	.053*	7	36
Mbuagbaw, 2016	RR (random model)	0.91 (0.71, 1.18)	0.89 (0.62, 1.27)	.500	.520	43	50
Wang, 2016	RR (fixed model)	1.07 (0.82, 1.40)	1.03 (0.76, 1.42)	.610	.830	41	60
Derry, 2017	RD (fixed model)	0.04 (-0.02, 0.09)	0.02 (-0.06, 0.10)	.210	.640	0	45
Knightly, 2017	RD (fixed model)	-0.01 (-0.05, 0.03)	0.02 (-0.06, 0.09)	.540	.690	0	0

Shen, 2017	RR (fixed model)	0.94 (0.68, 1.19)	0.76 (0.37, 1.57)	.690	.460	2	35
Tickell-Painter, 2017	RR (fixed model)	0.70 (0.14, 3.53)	0.94 (0.13, 6.62)	.660	.950	0	0
Verbeeck, 2017	RR (fixed model)	1.20 (0.35, 4.10)	0.20 (0.01, 4.01)	.770	.290	49	69
Zaman, 2017	RR (fixed model)	0.15 (0.06, 0.39)	0.12 (0.02, 0.92)	< .001	.040	0	0
Gallos, 2018	RR (random model)	0.62 (0.14, 2.74)	0.62 (0.14, 2.74)	.530	.530	0	0
Mehta, 2018	Peto OR (fixed model)	5.66 (3.62, 8.84)	4.85 (3.01, 7.81)	< .001	< .001	0	0
Panebianco, 2018	RR (fixed model)	0.19 (0.46,0.93)	0.81 (0.53, 1.23)	.041	.330*	23	32
Romantsik, 2018	RR (fixed model)	8.35 (5.31, 13.12)	7.05 (4.43, 11.21)	< .001	< .001	29	18
Sliwka, 2018	RR (random model)	0.96 (0.50, 1.83)	0.90 (0.33, 2.40)	.900	.830	0	-
Gagyor, 2019	RR (fixed model)	1.16 (0.83, 1.63)	1.08 (0.69, 1.67)	.390	.740	0	0
Solomon, 2019	RR (fixed model)	0.40 (0.36, 0.441)	0.42 (0.38, 0.46)	< .001	< .001	82	4
Toupin April, 2019	RR (fixed model)	0.65 (2.20, 3.19)	3.25 (2.27, 4.64)	< .001	< .001	45	17

Peto OR: Peto odds ratio; RR: risk ratio; RD: risk difference.

*: The conclusion changed from statistical significance to no statistical significance.

Table S4. Impact of excluding the trials with FCOI on the conclusion of serious adverse events.

Study	Statistical Method	Effect Estimate		*P* value		I^2	
		Original	Recalculated	Original	Recalculated	Original	Recalculated
Rudic, 2012	RR (fixed model)	0.87 (0.68, 1.12)	0.71 (0.46, 1.10)	.280	.120	23	0
Zani, 2014	RR (fixed model)	1.20 (0.59, 2.42)	1.29 (0.56, 7.00)	.620	.490	0	0
Sherlock, 2015	RR (fixed model)	3.04 (0.32, 28.99)	3.05 (0.13, 74.10)	.330	.490	0	-
Mbuagbaw, 2016	RR (random model)	0.91 (0.71, 1.18)	0.89 (0.62, 1.27)	.500	.520	43	50
Tickell-Painter, 2017	RR (fixed model)	0.70 (0.14, 3.53)	0.98 (0.20, 4.79)	.660	.980	0	0

FCOIs: financial conflict of interests;RR: risk ratio.

Table S5. Impact of trials without reported FCOIs on the conclusion of effectiveness.

Study	Statistical Method	Effect Estimate		*P* value		I^2	
		Original	Recalculated	Original	Recalculated	Original	Recalculated
Brand, 2010	RR (fixed model)	0.57 (0.34, 0.94)	0.58 (0.25, 1.35)	.028	.210	0	33
Marshall, 2010	OR (random model)	1.65 (1.11, 2.45)	0.13 (0.02, 0.24)	.010	.020	0	0
Chauhan, 2012	RR (random model)	1.51 (1.17, 1.96)	1.89 (1.59, 2.34)	.002	.009	42	28
Nair, 2012	MD (random model)	-0.36 (-4.09, 3.38)	-6.96 (-18.78, 4.87)	.850	.250	31	71
Brown, 2013	MD (random model)	-3.66 (-5.28, -2.05)	-2.66 (-7.99, -2.67)	< .001	< .001	60	83
Dold, 2015	RR (random model)	0.93 (0.87, 1.00)	0.99 (0.58, 1.69)	.046	.097*	0	66
Sherlock, 2015	RR (fixed model)	2.25 (1.50, 3.39)	1.98 (1.26, 3.09)	< .001	.003	79	0
Conway, 2016	RR (random model)	0.94 (0.48, 1.84)	1.03 (0.38, 2.82)	.850	.950	71	81
Wang, 2016	RR (fixed model)	0.69 (0.62, 0.77	0.61 (0.51, 0.74)	< .001	< .001	15	0
Zhu, 2016	MD (fixed model)	-0.14 (-0.20, -0.08)	-0.28 (-0.41, -0.15)	< .001	< .001	68	0
Robertson, 2017	Peto OR (fixed model)	0.69 (0.56, 0.93)	0.56 (0.25, 0.97)	.041	.038	0	0
Tickell-Painter, 2017	RR (random model)	0.09 (0.04, 0.19)	0.02 (0.01, 0.07)	< .001	< .001	53	67
Zaman, 2017	RR (fixed model)	1.13 (0.83, 1.54)	1.07 (0.56, 2.03)	.450	.850	18	0
Gallos, 2018	RR (random model)	1.26 (1.11, 1.43)	1.29 (1.13, 1.47)	< .001	.001	0	0
Mehta, 2018	MD (fixed model)	-2.29 (-6.03, 1.45)	-3.11 (-7.36, 1.15)	.230	.150	0	71
Panebianco, 2018	RR (fixed model)	1.89 (1.40, 2.55)	1.94 (1.42, 2.64)	< .001	< .001	0	0
Gagyor, 2019	RR (random model)	0.54 (0.38, 0.77)	0.50 (0.25, 0.97)	< .001	.040	40	68
Solomon, 2019	RR (fixed model)	0.57 (0.39, 0.84)	0.59 (0.38, 0.91)	.005	.008	23	0
Toupin April, 2019	SMD (fixed model)	-0.25 (-0.23, -0.19)	-0.45 (-0.67, -0.02)	< .001	< .001	0	0

FCOIs: financial conflict of interests; RR: risk ratio; OR: odds ratio; MD: mean difference; SMD: standardized mean difference.

*: The conclusion changed from statistical significance to no statistical significance.

Table S6. Impact of trials without reported FCOIs on the conclusion of major adverse events.

Study	Statistical Method	Effect Estimate		*P* value		I^2	
		Original	Recalculated	Original	Recalculated	Original	Recalculated
Lodi, 2012	RR (random model)	1.98 (1.10, 3.59)	3.38 (0.19, 61.03)	.023	.410*	11	-
Flenady, 2013	RR (fixed model)	1.32 (0.92, 1.89)	1.53 (0.91, 2.57)	.130	.110	0	38
Prasad, 2014	RR (fixed model)	0.43 (0.30, 0.62)	0.43 (0.30, 0.62)	< .001	< .001	48	-
Clarke, 2014	RR (fixed model)	0.91 (0.74, 1.12)	0.81 (0.63, 1.05)	.370	.110	0	0
Sherlock, 2015	RR (fixed model)	1.09 (0.95, 1.26)	1.03 (0.86, 1.23)	.220	.770	54	51
Nielsen, 2016	RR (random model)	1.10 (0.64, 1.91)	1.13 (0.64, 1.97)	.720	.680	52	55
Wang, 2016	RR (fixed model)	1.07 (0.82, 1.40)	1.03 (0.76, 1.42)	.610	.830	41	60
Knightly, 2017	RD (fixed model)	-0.01 (-0.05, 0.03)	0.02 (-0.06, 0.09)	.540	.690	0	21
Shen, 2017	RR (fixed model)	0.94 (0.68, 1.19)	0.76 (0.37, 1.57)	.690	.460	2	35
Tickell-Painter, 2017	RR (fixed model)	0.70 (0.14, 3.53)	0.31 (0.01, 7.54)	.660	.470	0	-
Zaman, 2017	RR (fixed model)	0.15 (0.06, 0.39)	0.12 (0.02, 0.92)	< .001	.040	0	0
Mehta, 2018	Peto OR (fixed model)	5.66 (3.62, 8.84)	4.39 (1.45, 13.23)	< .001	.009	0	0
Panebianco, 2018	RR (fixed model)	0.79 (0.46, 1.35)	0.81 (0.53, 1.23)	.250	.330	23	32
Gagyor, 2019	RR (fixed model)	1.16 (0.83, 1.63)	1.00 (0.36, 2.79)	.390	1.000	0	-
Solomon, 2019	RR (fixed model)	0.40 (0.36, 0.441)	0.44 (0.36, 0.53)	< .001	< .001	82	0
Toupin April, 2019	RR (fixed model)	0.65 (2.20, 3.19)	3.25 (2.27, 4.64)	< .001	< .001	45	44

FCOIs: financial conflict of interests; RR: risk ratio; RD: risk difference; Peto odds ratio;

*: The conclusion changed from statistical significance to no statistical significance.

Table S7. Impact of trials without reported FCOIs on the conclusion of major adverse events.

Study	Statistical Method	Effect Estimate		*P* value		I^2	
		Original	Recalculated	Original	Recalculated	Original	Recalculated
Rudic, 2012	Rudic, 2012	RR (fixed model)	0.87 (0.68, 1.12)	.280	.120	23	0
Sherlock, 2015	Sherlock, 2015	RR (fixed model)	3.04 (0.32, 28.99)	.330	.560	0	5
Tickell-Painter, 2017	Tickell-Painter, 2017	RR (fixed model)	0.70 (0.14, 3.53)	.660	.810	0	17

FCOIs: financial conflict of interests; RR: risk ratio.

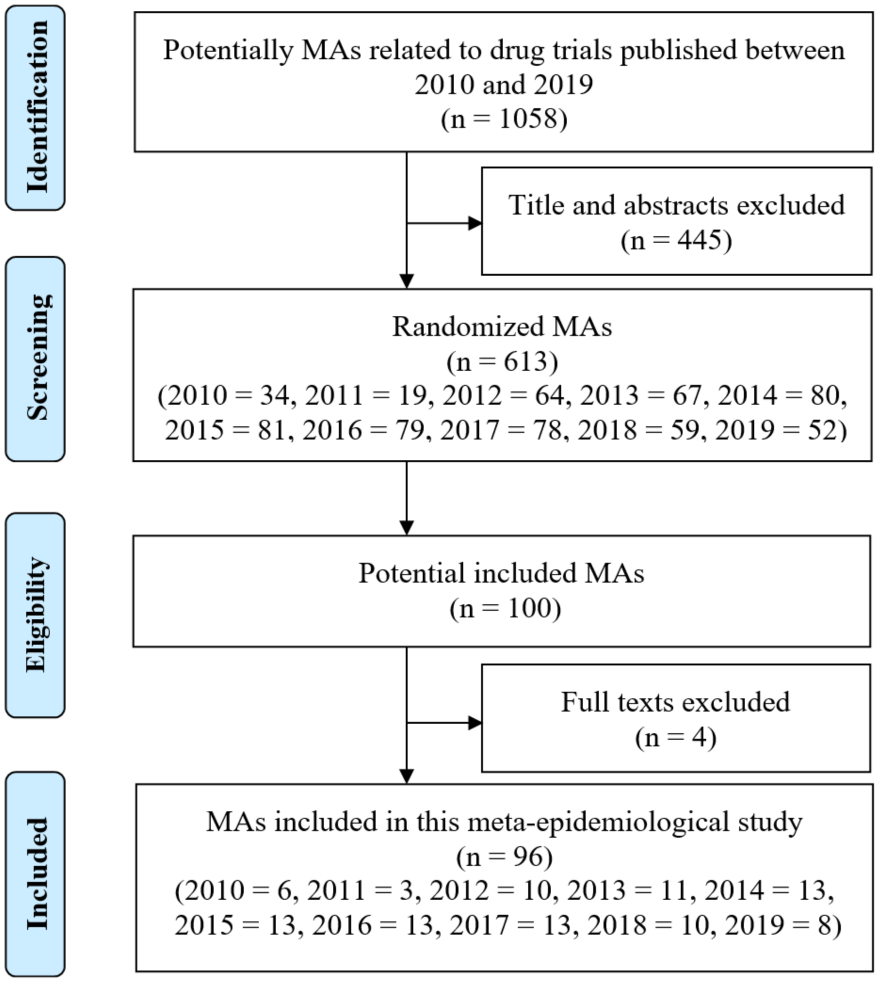

Figure S1. Study flow diagram.
